# The Overview of Human Localization and Vital Sign Signal Measurement Using Handheld IR-UWB Through-Wall Radar

**DOI:** 10.3390/s21020402

**Published:** 2021-01-08

**Authors:** Degui Yang, Zhengliang Zhu, Junchao Zhang, Buge Liang

**Affiliations:** 1 School of Aeronautics and Astronautics, Central South University, Changsha 410083, China; degui.yang@csu.edu.cn (D.Y.); junchaozhang@csu.edu.cn (J.Z.); liangbuge@csu.edu.cn (B.L.); 2 Key Laboratory of Underwater Acoustic Communication and Marine Information Technology of the Ministry of Education, Xiamen University, Xiamen 361005, China; 3 College of Ocean and Earth Sciences, Xiamen University, Xiamen 361005, China

**Keywords:** impulse radio (IR), ultra-wideband (UWB), through-wall radar, human information, vital sign signal, human location

## Abstract

Obtaining information (e.g., position, respiration, and heartbeat rates) on humans located behind opaque and non-metallic obstacles (e.g., walls and wood) has prompted the development of non-invasive remote sensing technologies. Due to its excellent features like high penetration ability, short blind area, fine-range resolution, high environment adoption capabilities, low cost and power consumption, and simple hardware design, impulse radio ultra-wideband (IR-UWB) through-wall radar has become the mainstream primary application radar used for the non-invasive remote sensing. IR-UWB through-wall radar has been developed for nearly 40 years, and various hardware compositions, deployment methods, and signal processing algorithms have been introduced by many scholars. The purpose of these proposed approaches is to obtain human information more accurately and quickly. In this paper, we focus on IR-UWB through-wall radar and introduce the key advances in system design and deployment, human detection theory, and signal processing algorithms, such as human vital sign signal measurement methods and moving human localization. Meanwhile, we discuss the engineering pre-processing methods of IR-UWB through-wall radar. The lasts research progress in the field is also presented. Based on this progress, the conclusions and the development directions of the IR-UWB through-wall radar in the future are also preliminarily forecasted.

## 1. Introduction

Obtaining information (e.g., position, respiration, and heartbeat rate) on humans behind opaque and non-metallic obstacles is an interesting project. By using a low-center frequency (400 Megahertz (MHz)-5 Gigahertz (GHz)) electromagnetic wave, radar can detect a human behind a wall with a non-contact and non-damaging method, called through-wall radar. Compared to other sensors (such as acoustic and infrared sensors), radar protects privacy and works well in different lighting conditions, even in darkness. In 2002, the FCC greenlit the civil use of ultra-wideband (UWB) technology and defined a UWB signal: The bandwidth of the transmitted signal must be 25% greater than the central frequency called the Ultra-wideband (UWB) signal [1]. Generally, through-wall radar using a UWB signal can penetrate an obstacle better than its alternatives due to the wideband effect. The UWB signal has several key advantages than over other signals: (1) a high range resolution; (2) a large bandwidth that can reduce the influence of obstacles in the signal and enhance the target recognition ability; (3) its immunity to multipath interference, making it suitable for indoor or complex environments.

The research on UWB through-wall radar can be divided into two categories (human information detection and information acquisition of internal structures of obstacles) according to the different detection environments and purposes. The latter methods are similar to those for ground penetrating radar (GPR), so the related processing methods for GPR are suitable for through-wall radar. Under the framework of UWB signals, the signal forms mainly include impulse radio (IR), random noise wave (M-sequence), frequency-modulated continuous wave (FMCW), and stepped frequency continuous wave (SFCW) [2]. Although there are many categories of UWB through-wall radar, IR-UWB through-wall radar offers excellent performance [3]: (1) Its data acquisition is faster than that of SFCW-UWB through-wall radar; (2) it uses a simple design for its transmitter and receiver; and (3) it operates at low power levels, giving it a long endurance time. Therefore, IR-UWB through-wall radar has promising prospects and applications for anti-terrorism efforts, post disaster search and rescue, and smart homes.

This paper presents a comprehensive review of the latest research on IR-UWB through-wall radar. Unlike other review articles on UWB radar [4,5,6,7,8], in this paper, we focused on the common technological methods used in the UWB through-wall radar and introduced the generalized IR-UWB radar components and their development history. The rest of this paper is organized as follows. In Section 2, the general composition of IR-UWB through-wall radar and the classical human UWB signal model are introduced. In Section 3, based on the engineering experience, we summarize the preprocessing algorithms for IR-UWB through-wall radar. In Section 4, we present the contributions of other researchers regarding the vital sign signal measurements of stationary humans and the locations of moving humans. Finally, conclusions and future prospects are provided in Section 5.

## 2. The Development of the Human Target Signal Model of IR-UWB Through-Wall Radar

### 2.1. The Development of IR-UWB Through-Wall Radar

**Figure 1:**
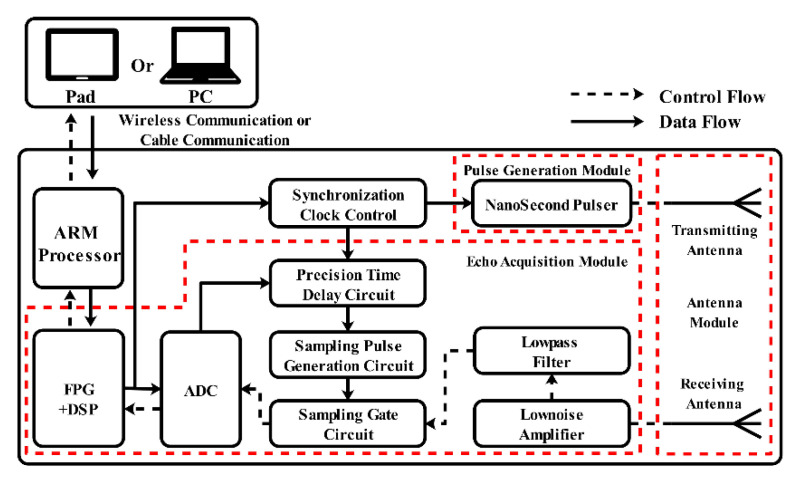
IR-UWB system block diagram.

**Figure 2:**
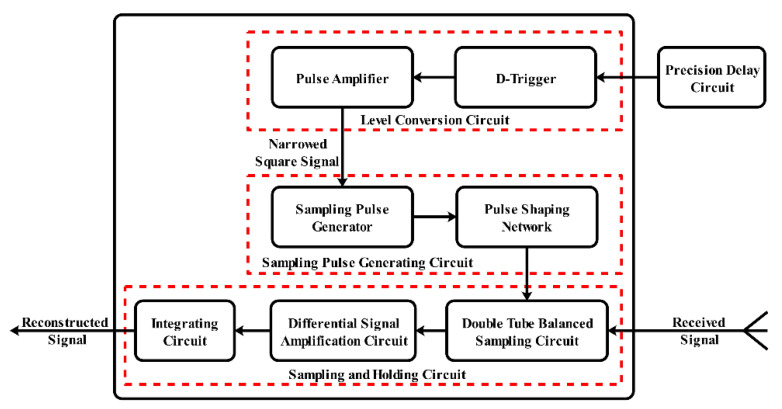
The block diagram of the echo signal acquisition module.

The study of IR-UWB through-wall radar system began around 1980, more than 40 years ago. Generally speaking, the hardware of IR-UWB through-wall radar systems is more concise than that of other through-wall radar solutions, like FMCW and SFCW through-wall radar [3], which consist of three parts: an impulse signal generation module, an echo signal acquisition module, and a data processing communication module. The corresponding system block diagram is shown in Figure 1. The design of the impulse signal generation module focuses on an all-solid state with high stability and a high repetition rate. This design can be implemented by using a field effect transistor (FET) or step recovery diode (SRD) as the core components. Because the impulse signal needs a high-speed analog-digital converter (ADC) for sampling, it is not conductive to radar system engineering implementations. As the transmitted signal is a periodic repetitive signal, the design scheme of the echo signal acquisition module based on equivalent sampling technology can be completed using a Field Programmable Gate Array (FPGA) and a low-rate analog-digital converter. The precision delay circuit is controlled by FPGA and generates the sequence delay square signal. This signal is processed by narrowing and amplifying it to force the sampling generation circuit to trigger the sampling gate circuit. Figure 2 provides more details about this module. The data processing communication module is responsible for the dual flow of data. When the detection data are transmitted to the module, users also can issue work instructions. Usually, the module uses a cable or wireless method (e.g., Wi-Fi, Zigbee, or Bluetooth) to interact with users.

There are some representative research institutions and organizations that focus on the design and implementation of IR-UWB through-wall radar systems: Timedomain Co., Ltd. (Huntsville, AL, USA); the Cambridge consultants company (Cambridge, Cambridgeshire, UK); the Camero-Tech company of Israel (ISR); and the design bureau of the experimental works of Russia (RUS). In China (CHN), there are many enterprises that have established cooperative relationships with universities and carried out considerable pioneering research, such as Xi’an Biken technology development Co., Ltd. (Xi’an, China), the Novasky Electronic technology Co. Ltd. (Changsha, China), the China Research Institute of Radio wave Propagation; and Beijing LSJ technology development Co., Ltd. (Beijing, China).

In 1998 and 2001, the Timedomain company [9,10,11] developed an IR-UWB through-wall radar based on its self-developed UWB integrated circuit named PulseOn; the corresponding models are RadarVision1000 and RadarVision2000. RadarVision1000 has a large transmitted signal bandwidth of 1.4 GHz. Its center frequency is 2 GHz, and its pulse repetition frequency (PRF) is 5 MHz. This radar system can reach 4-inch (10.16 cm (cm)) range resolution. Compared with the former, RadarVision2000 can realize 2-dimensional imaging of the target focus in any detection area. Its PRF can reach 10 MHz, its center frequency is 2.4 GHz, and its transmitted signal bandwidth is around 0.7 GHz. The transmitted power of this radar system is as low as 50 milliwatts (mW). The detection distance is more than 20 m (m) when the thickness of the concrete wall is about 20 cm. Following RadarVision2000, the Timedomain company launched a military version named SoliderVision2000a1 (SV2000A1) to meet the operational needs of the army in densely populated areas. This device can be used by soldiers to perform handheld work [10,12]. SV2000A1 was used by the U.S. Army in its urban operations in Iraq during 2003. This radar system uses 11 pairs of receiving and transmitting spiral antennas. The center frequency of its transmitted signal is 2 GHz, and its bandwidth is also 2 GHz; its transmitted power is 1.5 mW. The short pulse transmitted by each pair of antennas is precisely encoded by a timing chip, and target movement is detected by detecting the energy changes of adjacent returns. SV2000A1 has a detection field of 60 degrees in the horizontal direction and 45 degrees in the vertical direction. Its optional detection range is 3, 5, and 10 m, and it can operate under multi-mode detection according to the speed of the moving target. At present, the products of the Timedomain company include PulsOn series UWB radar modules. The PulseOn440 UWB radar has a size of millimeters (mm). The bandwidth range of this module is 3.1–4.8 GHz, and its center frequency is 4.3 GHz. It can work in single, double, and multi station network. The measurement accuracy of this device is 1 cm on open ground, and its indoor test accuracy is better than 1 m under the conditions of non-line of sight (NLOS).

In 2006, The British Cambridge consultant company developed a second version of the portable radar interior space monitor 200 system (Prism-200) using coherent short pulses as the transmitting signals with a bandwidth range of 1.6–2.2 GHz with a center frequency of 1.9 GHz. Prism-200 can acquire and track the 3-dimensional spatial position and speed of a target hidden behind an obstacle. The spatial resolution of this system is 30 cm, and its maximum detection range is 15 m [13].

Israel Camero-Tech company began to develop the XaverTM series IR-UWB through-wall radar system in 2004, which is suitable for penetrating detection through wood, brick walls, concrete walls, or other non-metal obstacles. XaverTM-800 is the most advanced through-wall radar system in this series. It uses a 24-element antenna array to perform 3-dimensional imaging and tracking of its detected targets. The paper [14] presents this radar system and its operational results. XaverTM-800 has a horizontal and vertical detection angle of 80 degrees with a bandwidth of 7 GHz. The system’s range resolution is 3 cm, and its azimuth resolution is 30 cm when the detection range is 8 m. XaverTM-100 is a handheld portable through-wall radar system. XaverTM-400 is an upgraded version based on XaverTM-100, whose running frequency range is 3–10 GHz. Its farthest detection range is 20 m. The range resolution of this system is 5 cm. In 2016, Camero-Tech developed a remote-controlled system termed XaverNETTM for the coordinated detection of XaverTM-100 and XaverTM-400. This remote-control system uses the Zigbee data transmission protocol and supports up to four radar system for distributed detection. The relevant area is constructed with multiple perspectives, which can eliminate the blind areas to a certain extent. Meanwhile, the collaborative system has a data playback function for replay analysis after the end of a task.

In China, under the support of funds from relevant departments, Xi’an Biken company and the Fourth Military Medical University (FMMU) jointly developed a more advanced wall-penetrating life detection IR-UWB radar like the PoliceVision series called the SJ series [6]. Among them, the SJ-3000 life detection radar participated in the ‘2006 Asia Pacific Earthquake Drill’ exercise and was put into use in the 2008 Wenchuan Earthquake in Sichuan province. At present, the newest product model of Biken company is the SJ6000+ life detection radar which can penetrate walls with a thickness of 42 cm. This radar system has the ability to detect the breathing signal of a stationary human within 18 m and a moving human target within 27 m. The latest research achievement of FMMU is an IR-UWB multi-channel radar with a center frequency of 400 MHz. This radar system transceiver’s antennas have multiple degrees of freedom due to their special mechanical design [15]. In this way, the position and detection direction of each transceiver element can be adjusted according to the detection scenery, making this device applicable to complex environments; detailed information can be found in the latest paper [15].

In 2005, The National University of Defense Technology (NUDT) developed a through-wall radar system named RadarEye with the UHF and L frequency bands [16]. The transmitted signal used in this radar is a bipolar short pulse signal without a carrier frequency, and the effective detection range is 3–5 m. The RadarEye system produces low electromagnetic interference in the surrounding environment, making it suitable for deployment in sensitive areas. The Novasky Electronic technology company [17] is a young start-up company developing UWB through-wall radar solutions in China. It developed its CE/CEM/DN/YSR series products in cooperation with NUDT. The CE-200 IR-UWB through-wall radar uses multiple input/multiple output technology (MIMO), is able to detect 3–10 human targets at the same time, and has a range resolution of 30 cm. The DN-IV life detection radar has a medium compensation ability in a variety of environments. The YSR30 radar system was developed for special environments like mines and can obtain the vital signs of a fully stationary human target.

The Beijing LSJ technology development company and the team of Central South University (CSU) established a university–industry cooperation relationship, and the resulting IR-UWB through-wall radar shows excellent performance in actual operations [18]. The LSJ radar uses a dielectric coupling enhanced antenna which improves the device’s anti-interference capabilities and has the ability to estimate the dielectric constant from environment media. After the collapse of the Xinjia Hotel in Quanzhou City, Fujian Province on 7 March 2020 [19], the LSJ through-wall radar successfully detected three people on March 9 and March 10.

In summary, existing IR-UWB through-wall radar has the following features: (1) It has the ability to capture information on multiple moving human targets and multiple micro-moving human targets and (2) it has no fixed mode for deployment of its transceiver antennas. Most radar systems use compact transceiver antennas to reduce the volume of the radar, but this may cause an artifacting problem due to the short radar aperture.

### 2.2. The Human Return Signal Model of the IR-UWB Through-Wall Radar

In past studies, many papers build models to describe human return signals. In Fei et al. [20] the human target echo of a UWB through-wall radar was modeled in the time-domain. The received signal was complex and could not be understood by the simple delay and doppler frequency of the transmitting signal but required the superposition of different time delays of multiple scattering points of the whole target. After the first filter of the wall, the signal transmitted by the radar penetrated the wall and irradiated the target, then the radar wave penetrated the wall a second time and was received by the receiver together with other noises. A specular multipath model for UWB radar was introduced in [21,22]. The return signal included a multi-path component because the return wave came from different human body parts at different times with various amplitudes. These different scattering pathways should be considered as multipath components of the UWB radar’s received signal. Therefore, the time-varying UWB multipath channel model can be used to describe the human return signal [23]:

(1) $$h(t,\tau )=\sum_{j=1}^{L}a_{j}(t)w_{j}(\tau -\tau_{j}(t))$$

where τ is the time delay, and t is the UWB device’s elapsed time. The channel model is represented as the superposition of the L strongest scattering path. These paths describe the response at time t. $a_{j}$, $\tau_{j}$ denote the amplitude and the time-of-arrival (TOA) of path j, respectively. $w_{j}(\cdot )$ is the waveform of path j, which can be approximated by the specular multi-path channel model, namely $w_{j}(\cdot )=\delta (\cdot )$, where δ(⋅) is the Dirac delta function [24]. This simplification ensures that the mono-static UWB radar runs in the presence of real-time processing.

**Figure 3:**
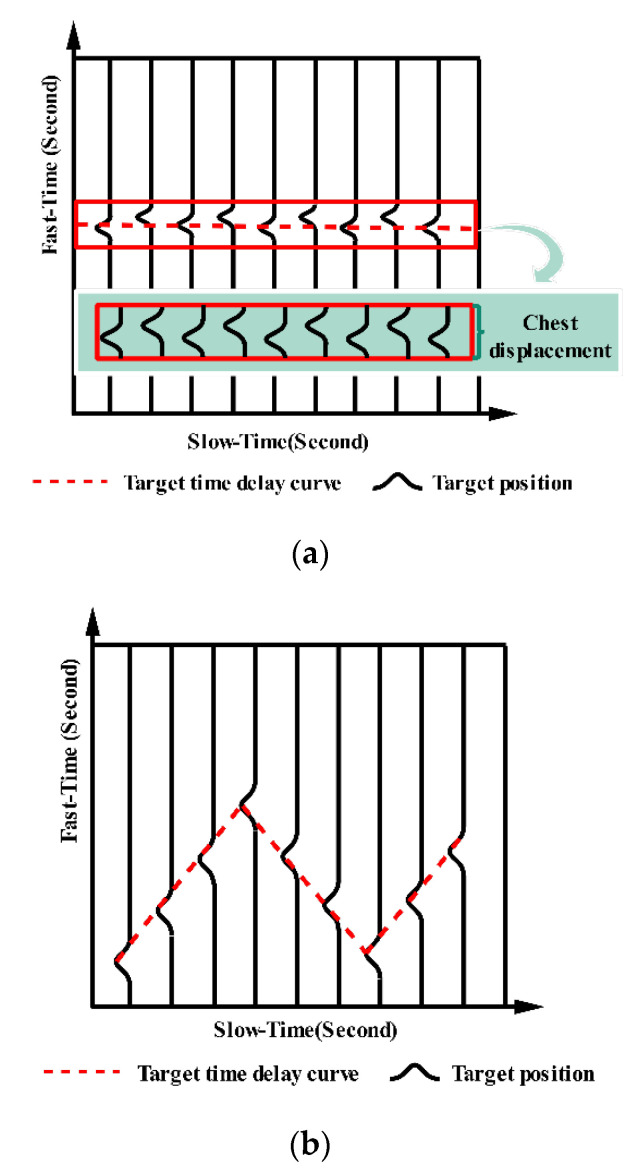
The signal model illustration of a human target. (**a**) the signal model of a human standing still; (**b**) the signal model of a moving human.

Therefore, the UWB radar’s received signal can be represented as follows:

(2) $$r(t)\approx \sum_{j=1}^{L}b_{j}(t)p_{j}(\tau -\tau_{j}(t))$$

where r(t)=h(t,τ)⊗p(τ) and ⊗ means the convolution operation. p(τ) is the impulse signal transmitted by the radar transmitter. $b_{j}(t)$ represents the signal amplitude factor. $\tau_{j}(t)$ refers to the path j’s propagation delay at time t from the transmitter to the receiver. Discretize (2) along both the slow-time direction and the fast-time direction (the slow-time represents the received pulse number and the fast-time represents the total sampling points that can be converted to distance [25]). The radar received signal matrix can then be obtained and some relevant information can be extracted (the specific method will be introduced in Section 5). Considering only one human target in the scene, we can solve the human position using TOA $\tau_{j}(t)$. Meanwhile, along the slow-time direction, the human target’s respiratory and heartbeat rates can be analyzed. Figure 3 illustrates the human signal model (in Figure 3b, we magnified the displacement of the chest cavity intentionally).

**Figure 4:**
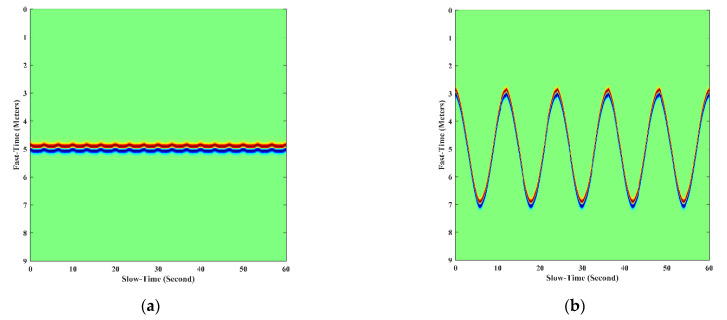
The simulation signals of human target in different motion states. (**a**) human standing still; (**b**) human walking back and forth.

Based on the UWB radar received signal model (2), the return signal of a stationary human was simulated in [26]. In this paper, the return signal of a moving human was simulated. In these simulations, we used the following assumptions: The signal transmitted by the radar had no attenuation during propagation, and the transmitted signal was a Gaussian pulse. The transceiver antenna of the radar remained under ideal conditions. The human scatter only considered the torso and the displacement of the chest. The simulation scenarios were as follows. The human target was standing still with a simulated respiratory frequency of 0.3 Hz, and his/her chest displacement was 0.05 m. The moving human was walking back and forth along the radar line of sight radially. The simulation running time was 60 s. Figure 4 shows the simulation signals.

## 3. The Pre-Processing of Radar Signals before Human Information Is Obtained

Based on the human target detection theory in Section 2, the radar received signals with a complex composition, which was necessary to remove clutter (for example, the wall reflection wave, antenna direct coupling wave, background echoes, and other noises) as much as possible in the low computational method. For the target echo obtained after clutter suppression, the signal amplitude was relatively weak, and appropriate signal amplitude enhancement was needed. The most common clutter removal methods and signal enhancement algorithms are described below. Meanwhile, we also introduced the wall parameter estimation method, which uses the return signal and the reference time zero search method.

### 3.1. The Clutter Removal Methods

#### 3.1.1. Pulse Cancelling

In the through-wall radar detection scene, the reflected signals of the target contain high-frequency components in the frequency domain. When the target maintains a large amplitude motion, the high-frequency component is greater. The pulse cancellation method has the function of a high pass filter in the frequency domain; therefore, this method can remove clutter to some extent. Here, $r_{t+1}$, $r_{t}$ represents the radar received signal at t+1, t time. Then, the human reflected echoes $z_{t}$ can be expressed as

(3) $$z_{t}=r_{t+1}-r_{t}$$

This method achieves the effect of linear filtering by differentially processing the adjacent return signals, thus realizing the removal of clutter components [27]. This method can remove most of the background clutter in the detection scene, but when the human target displacement is small, this method has difficulty in retaining the human echoes.

#### 3.1.2. Cumulative Average Background Cancellation

The accumulated average background cancellation method is different from the pulse cancellation method. It first estimates the background signal $y_{t+1}$ at t+1 time by the mean value of historical echoes:

(4) $$y_{t+1}=mean\{r_{1},r_{2},\ldots ,r_{t}\}$$

where $y_{t+1}$ represents the background signal at t+1 time, $r_{1},r_{2},\ldots ,r_{t}$ represents the historical radar received signals, and the mean{⋅} is the mean operation. Therefore, the human target signal can be expressed as follows:

(5) $$z_{t+1}=r_{t+1}-y_{t+1}$$

Theoretically speaking, the cumulative average background cancellation method is the most accurate background mean estimation algorithm. However, according to an analysis of Equation (5), this algorithm does not have the ability to use real-time processing. At the same time, it also has higher requirements for the storage capacity of the radar system. To give this algorithm online processing abilities and reduce its data storage capacity, researchers rewrote the Equation (5) as

(6) $$\begin{array}{ll} y_{t+1} & =mean\{r_{1},r_{2},\ldots ,r_{t}\}\\ & =(1-\frac{1}{t})y_{t}+\frac{1}{t}r_{t+1} \end{array}$$

In Equation (6), $y_{t+1}$ consists of two items: The first item is the estimated background signal at t time, and the second item is the received signal at t+1 time. With an accumulation of the detection time, the background estimation accuracy of the algorithm increases. However, when the background changes in the detection scene, the background update speed is slow because in this algorithm, the weight of each echo is at the same level, so the algorithm cannot focus on the latest echo data, which makes the current echo data contribute less to the whole background estimation signal.

#### 3.1.3. Exponentially Weighted Cancellation

The difference between the exponentially weighted cancellation method [28] and the cumulative average background cancellation method lies in its background signal estimation:

(7) $$\begin{array}{ll} y_{t+1} & =\alpha y_{t}+(1-\alpha )r_{t+1}\\ & =(1-\alpha )(r_{t+1}+\alpha r_{t}+\cdots +\alpha^{2}r_{2})+\alpha^{t}y_{1} \end{array}$$

where α is the exponentially weighted factor, which determines the stability of the estimated background signal. In Equation (7), the estimated background signal using this method is the same as that in (4). Therefore, this method has the features of both previous methods. Using the weighting factor, the high frequency composition is smoothed. Meanwhile, this method can adopt variation in the environment. As the detection time passes, the weight of the current received signal becomes larger than that of the earlier echoes. Thus, this method can update the background information dynamically.

In summary, the purpose of the clutter removal method is to retain the interesting target echoes and remove the irrelevant scattering signals as much as possible. The corresponding core solution depends on real-time estimation of the background signal. The methods mentioned above are widely used because of their low complexity, but their performance is related to the human target’s trajectory. There are many existing methods based on spatial filtering, subspace projection, compressed sensing, etc. [29,30,31,32,33]. Improved exponentially weighted cancellation based on the least mean square can better solve the problem of target loss when the target remains in movement conversion, but its structure is more complex [34]. The method based on singular value decomposition has a weak coupling degree with the form of the target’s movement, but it requires a large amount of computation [35,36].

### 3.2. The Signal Enhancement Algorithms

In the traditional radar principle, the power of radar-received signals reflected by targets depends on the distance between the radar antenna and the target. UWB through-wall radar also has this property and must focus on the effect of the penetrating medium on its signal propagation. Especially in a multi-target tracking scenario, the reflection from the target located further from the radar is weaker than that near the target [37]. Meanwhile, the nearer target will shadow the relatively distant target [38], making it more challenging to detect the distant target. Many weak signal enhancement algorithms were proposed to solve this problem to some extent [37,39,40]. Generally, the principle of the weak signal enhancement algorithm involves equalizing the raw signal amplitudes by updating the time-varying gain coefficient, which can be expressed as follows:

(8) $$z_{E}(\tau )=z(\tau )\cdot \omega (\tau )$$

where $z_{E}(\tau )$ represents the enhanced signal, z(τ) denotes the raw signal without the enhancement algorithm, and ω(τ) is the time-varying gain coefficient series.

#### 3.2.1. Propagation Time Gain

The principle of the propagation time gain is based on the attenuation of signal strength with the propagation distance in the radar system. Namely, the time-varying coefficients can be expressed as a power term of the propagation time. Therefore, the expression of this algorithm is as follows:

(9) $$\omega (\tau )=\tau^{k},\;k=1,2,3,4.$$

where τ denotes the propagation time, and k denotes the power term coefficients.

#### 3.2.2. Advance Normalization

**Figure 5:**
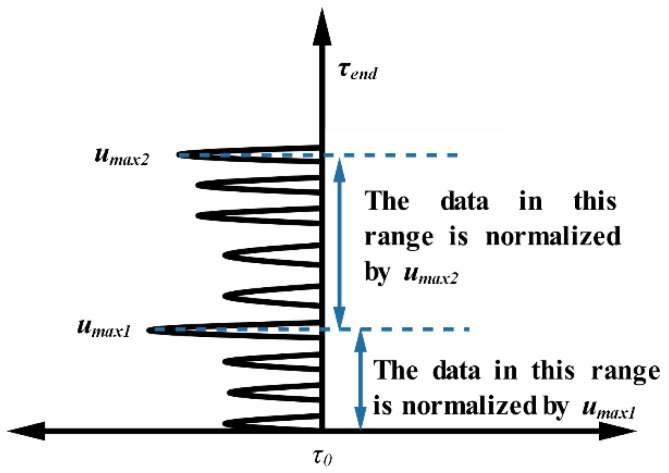
An illustration of advanced normalization.

The method proposed in [37] is called advanced normalization. Consider that the total interval of the received signal length is $\left[\tau_{0},\tau_{end}\right]$. First, the maximum value $u_{max1}$ of the total interval is calculated, and its index position is recorded as $\tau_{u1}$; then, the signal among $\left[\tau_{0},\tau_{u1}\right]$ is normalized based on $u_{max1}$. Second, the next largest value is found along the sub-interval of this signal $\left[\tau_{u1},\tau_{end}\right]$ and normalized in this interval. After reaching the index position at the end of the signal, the loop ends. This method is illustrated in Figure 5 with pseudo-code is shown in Algorithm 1.
**Algorithm 1** Advance1 normalization**Input:** Raw signal $z(t,\tau ),\;[\tau_{0},\tau_{end}]$**Output:** Enhanced signal $z_{E}(t,\tau )$$\tau_{Lmax}=0$$\tau_{Nmax}=\tau_{end}-1$**While**$\tau_{max}\leq \tau_{end}$ do   $\left[v_{max},\tau_{max}]=\underset{\tau \in [\tau_{Lmax},\tau_{end}]}{max}|(z(t,\tau )\right|$      **If**
$\tau_{Lmax}+\tau_{max}<\tau_{end}$
**then**          $\tau_{Nmax}=\tau_{Lmax}+\tau_{max}$      **Else**          $\tau_{Nmax}=\tau_{end}$      **End if**
      **For**
$\tau =\tau_{Lmax}\;\mathrm{to}\;\tau_{Nmax}$$z_{E}(t,\tau )=\frac{z(t,\tau )}{v_{max}}$      **End**      $\tau_{Lmax}=\tau_{Nmax}$**End while****End**


#### 3.2.3. Automatic Gain Control

The core idea of automatic gain control is to adjust the time-varying gain coefficients by feedback according to the features of the radar received signal. An automatic gain control (AGC) algorithm based on the signal power was mentioned in [37]. First, assume a sliding window length d and max gain $g_{max}$. Second, calculate the signal power under the given size window 2d+1 and compare it with $g_{max}$. The detailed calculation formulas are as follows:

(10) $$g(t,\tau_{i})=\frac{2d+1}{\sqrt{\sum_{k=i-d}^{i+d}z{\left(t,\tau_{i}\right)}^{2}}}$$

(11) $$g_{norm}(t,\tau_{i})=\frac{g(t,\tau_{i})}{\underset{\forall i}{min}\;g(t,\tau_{i})}$$

(12) $$g_{mask}(t,\tau_{i})=\{\begin{array}{ll} g_{max}, & g_{norm}(t,\tau_{i})>g_{max}\\ g_{norm}(t,\tau_{i}), & g_{norm}(t,\tau_{i})\leq g_{max} \end{array}$$

(13) $$z_{E}(t,\tau_{i})=g_{mask}(t,\tau_{i})z(t,\tau_{i})$$

where $g(t,\tau_{i})$ represents the variance based on the signal power, $g_{norm}(t,\tau_{i})$ denotes $g(t,\tau_{i})$ normalized by the minimal value, and $g_{max}$ is the gain mask used to enhance the raw signal z(t,τ).

This algorithm clearly requires the input of two parameters (i.e., the sliding window d and the max gain $g_{max}$), but the author doses not give any suggestions of how to select these two parameters. matGPR introduces similar signal-enhanced algorithms based on the application of GPR (e.g., standard AGC and Gaussian-tapered AGC) [41]. In matGPR, the time-varying signal level is computed by the signal root mean square (RMS) over a sliding time-window. During this time-window, the signal amplitude at the center of the window with respect to the RMS of the window scales. This process ensures that a low signal amplitude can be enhanced with respect to the high signal amplitude.

#### 3.2.4. Verification Based on Real-World Measured Data

**Figure 6:**
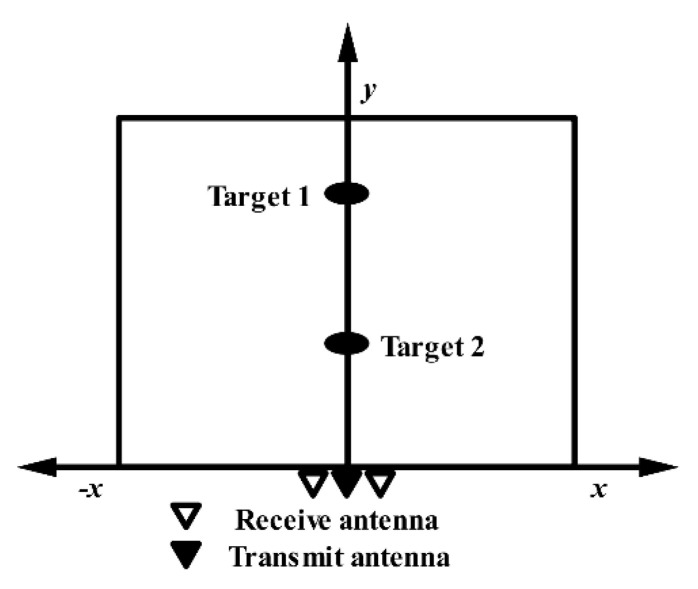
Actual experimental scene.

**Figure 7:**
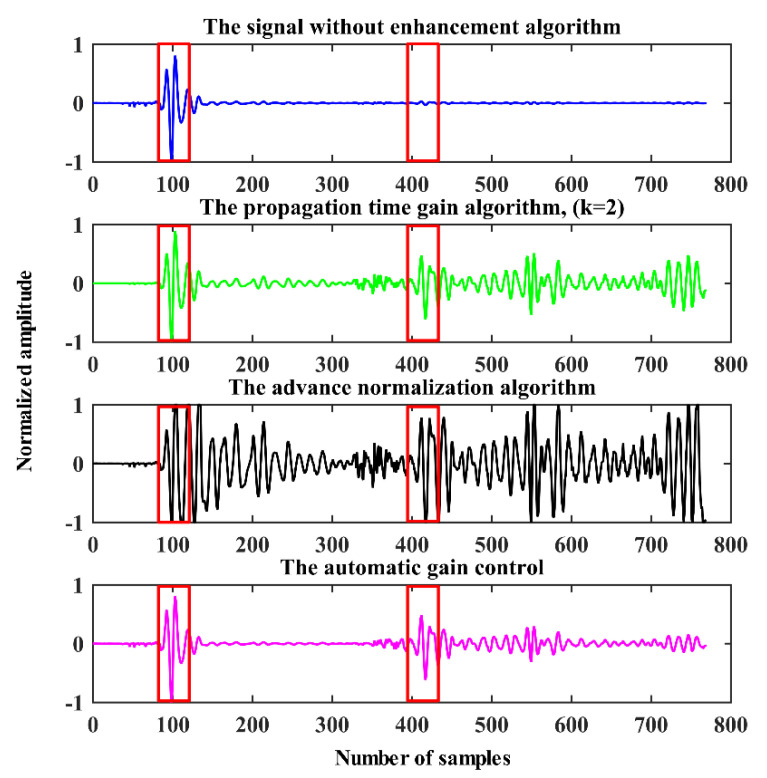
Actual experimental results.

To verify the performance of the above-mentioned signal enhancement algorithms, the radar system described in [42] was used for actual data acquisition. In the experiment, the human target linear motion trajectory is parallel to the radar detection line of sight, and the distance between the radar and human target is about 2–15 m. The experimental scene is shown in Figure 6, and the corresponding received signal with the enhanced algorithm is presented in Figure 7. The human target’s returned signal cluster is located near the 100th sampling point and the 420th sampling point. Figure 7 shows that the weak signal enhancement algorithm discussed in this section can amplify a human target’s return signal. In the signal without any enhanced algorithm, the human target signal cluster located near the 420th sampling point is overwhelmed by noise, and the enhancement algorithm effectively amplifies the return signal of the distant human target. However, although advanced normalization enhances the target signal, it also amplifies the noise signal amplitude, which is not conductive to the extraction of the target signal.

### 3.3. The Method for Reference True Time Zero Search and Time-Delay Calibration

**Figure 8:**
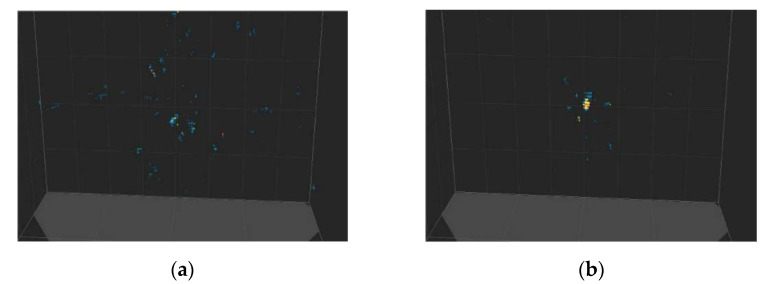
Comparison of the imaging results with/without time-delay calibration. (**a**) without time-delay calibration; (**b**) with time-delay calibration.

In an actual multi-channel through-wall radar system, the starting point of the echo signal is not the ‘zero time’ of radar. This is because the time delay of each piece of channel hardware is different and time-varying (due to temperature, voltage stability, and other physical parameters). To achieve the human localization of multi-channel radar, a zero-time search and time-delay calibration are required among the receiving channels. If not, the wrong position may be located or image defocusing may occur. Figure 8 presents a simple spherical object imaging result of XaverTM-800 with the time delay calibrated in different stages [14]. Figure 8 shows that time-delay calibration is significant for imaging results. In Figure 8a, the imaging result is defocused and cannot be considered as a spherical target.

**Figure 9:**
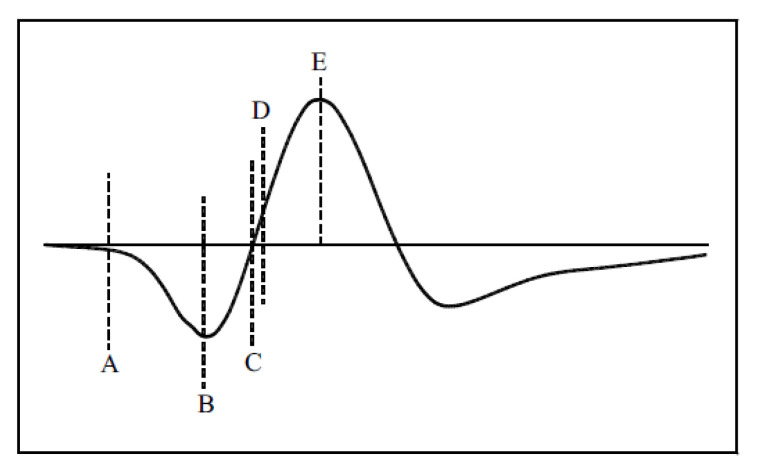
The reference zero-time position summary [43].

The work in [43] summarized its zero-time position findings, including (1) the first break point; (2) the first negative; (3) the zero amplitude point; (4) the mid-amplitude point; and (5) the first positive peak, as shown in Figure 9.

In this paper, we introduce a simple and efficient time-delay calibration method based on the first positive peak. The first positive peak point of the UWB echo signal can be regarded as the direct coupled wave position, as the wave amplitude is large, and the propagation range is the distance between the transmitting channel and receiving channel. Take a single input multiple output (SIMO) radar as an example, with 1 transmitter and N receivers (N > 0, N mod 2 = 0):

(14) $$t_{dir}=\frac{d_{trx}}{c}$$

(15) $$t^{\prime }{}_{dir}=t_{dir}+\Delta_{ch}$$

Assuming that there is no time delay in the channels, the first positive peak position can be calculated by (14), where c is the propagation velocity of electromagnetic waves, and $d_{trx}$ is the distance of the transmitting and receiving antenna. When considering the time delay in the channels, the direct coupling wave will appear delayed, and (14) should be rewritten as (15), where $\Delta_{ch}$ represents the time delay in the corresponding channel. Then, with these two equations, $\Delta_{ch}$ can be solved and used to calibrate the target position. The pseudo-code of the calibration method is shown in Algorithm 2.
**Algorithm 2** Multi-channel time delay calibration method based on the reference zero time**Input:**The distance of transmitting and receiving antenna set: $\left\{d_{t0r1},d_{t0r2},\ldots ,d_{t0rN}\right\}$The first positive peak position of each receive channel set: $\left\{{t^{\prime }}_{dir01},{t^{\prime }}_{dir02},\ldots ,{t^{\prime }}_{dir0N}\right\}$**Output:**The time delay with reference channel: $\left\{\epsilon_{21},\epsilon_{31},\ldots ,\epsilon_{N1}\right\}$.1. Setting the receive channel closest to the transmitting channel as the reference channel, namely the No.1 channel;2. Calculate the delay of each channel by (14) (15); the channel delay set can then be obtained: $\left\{\Delta_{ch1},\Delta_{ch2},\ldots ,\Delta_{chN}\right\}$;3. Calculate the delay shift between each channel and the reference channel: $\left\{\epsilon_{21},\epsilon_{31},\ldots ,\epsilon_{N1}\right\}$;   **For**
i=2 to N     **If**
$\epsilon_{i1}\leq 0$
**then**        Find the corresponding return signal, with zero padding from the starting position of the signal;        The padding number is $\epsilon_{i1}$;     **Else**        Find the corresponding return signal and discard the data from the starting position of the signal;        The discard number is $\epsilon_{i1}$;     **End if**   **End****End**


### 3.4. The Wall Parameter Estimation Method

During UWB through-wall radar sensing, the influence of the wall parameters like the dielectric constant and wall thickness of the signal should be considered. Due to the wall’s physical condition, the signal may experience attenuation, refraction, or diffraction, leading to a defocused target image and a wrong target position. In Wang et al. [44], the authors explored the effects of physical wall errors via simulations based on the wideband beamforming imaging method. The conclusions of this paper are as follows: When the dielectric constant is assumed to be known, if the estimated wall thickness is greater than the actual value, the azimuth and range coordinates of the target will both be smaller than the true position. However, with a known wall thickness, if the estimated dielectric constant is greater than the actual constant, then the target position will be closer to the radar. Currently, studies that focus on the wall parameter estimation can be classified into three categories: (1) direct measurement methods [45,46,47], (2) imaging feedback correction methods [44,48], and (3) time domain range profile extraction methods [49,50,51].

**Figure 10:**
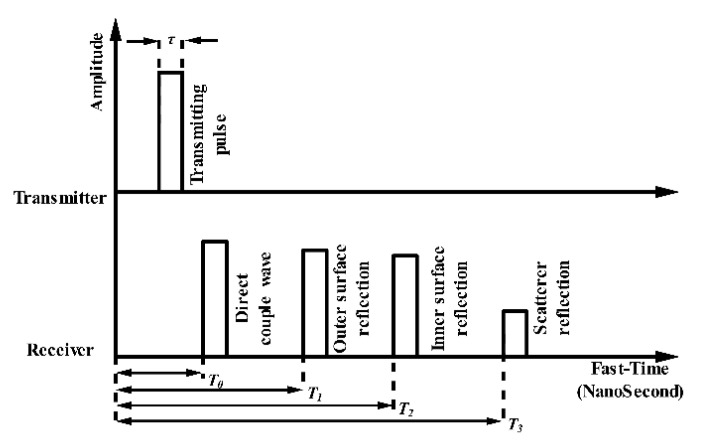
Wall reflection and signal components in the time domain.

In this paper, we review and introduce the progress of the third method. For practical application considerations, we only consider the distribution of the radar antennas on the same side. The UWB radar return signal contains abundant information on the wall, which can be extracted for wall parameter estimation. The signal components in the time domain are presented in Figure 10. Based on this figure, beside the direct coupling wave, the earliest echo is a reflection of the outer surface of the wall with time delay $T_{1}$, and the second return occurs from the inner surface wall with time delay $T_{2}$. The result is the scatterer reflection behind the wall with time delay $T_{3}$. The amplitudes of all reflections have certain attenuation compared to the transmitted pulse. Based on this phenomenon, a novel wall parameter estimation method was proposed in [51], which uses the time delay extracted from the signal range profile. Considering the essential connection between the observation process and the echo structure (namely, the precise extraction of the time delay), the proposed method acquires the wall thickness and permittivity exactly by using the numerical equation method and intersecting the searching curves. The simulation results demonstrate that the proposed method can reach a wall thickness error less than 1 cm and a dielectric constant error less than 1.

In the through-wall sensing problem, the non-linearity is reflected in the relationship between the backscatter and the wall parameters. In Zhang et al. [49], a well-trained support vector machine (SVM) was employed to solve the non-linearity problem. More specifically, the non-linearity problem can be abstracted as

(16) $$Y=f(E_{\mathrm{sca}})$$

where $Y=(y_{i}|y_{i}\in C)$ and $C=(d,\varepsilon_{r})$ denote the wall thickness and permittivity; $E_{\mathrm{sca}}=(E_{1},E_{2},\ldots )$ are the simulation return signals generated by the finite-difference time-domain (FDTD) method; and f(⋅) is the non-linearity unknown function trained by the SVM. If f(⋅) is well-obtained, then the uncertain wall parameters can be predicted quickly and accurately from the time domain signal range profile. The effectiveness of this method was demonstrated by repeated simulations in FDTD, where the estimation error for the wall thickness was less than 0.005 m, and the error for the wall permittivity was less than 0.1.

In Qu et al. [50], a method using a time-delay-only estimation strategy with a hybrid bistatic–monostatic measurement configuration was proposed to estimate the wall parameters. As Figure 10 shows, the reflected signals from the wall can be regarded as a template for the transmitted pulse with time-shifts and certain attenuation. This means that the propagation channel of the transmitted signal can be modeled as a sparse channel. Therefore, the sparse blind deconvolution algorithm is applied to precisely obtain the time delays of the return signals of the wall. With the time delays, the wall thickness, relative permittivity, and conductivity can be calculated. The simulation results based on the software package gprMax [52] verify the performance of this method compared with estimation of the signal parameters via the rotational invariance technique (ESPRIT) [53]. The proposed method offers excellent performance and has stronger noise tolerance. More detailed information can be found in Table 1 and Table 2.

## 4. Obtaining Human Information

### 4.1. Basic Theory of Human Localization

The human position algorithm is the core of IR-UWB through-wall radar. There are many location algorithms based on radio, such as direction of arrival (AOA), received signal strength indicator (RSSI), time difference of arrival (TDOA), and time of arrival (TOA). In this section, we introduce the ellipse cross positioning algorithm based on TOA, which is commonly used in IR-UWB through-wall radar [54]. This algorithm only requires the time delay information of targets, allowing it to fully use the extremely fine time resolution of the UWB signal. Since the algorithm requires at least two pairs of transmitting and receiving antennas, the following is a brief description of this algorithm based on a one-transmitter and two-receiver IR-UWB through-wall radar.

**Figure 11:**
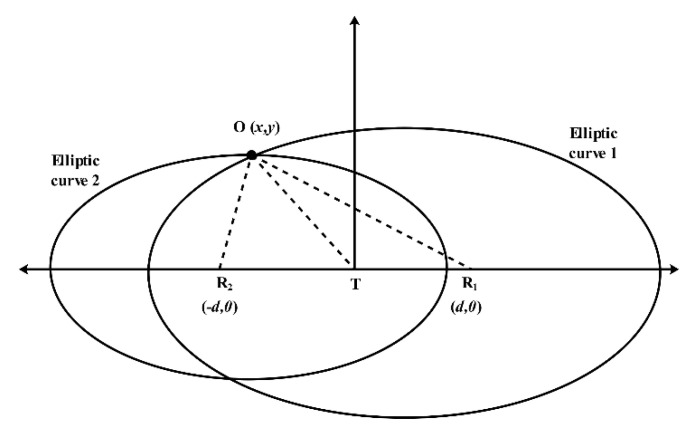
An illustration of the ellipse cross positioning algorithm (without obstacles between the radar and target).

Assuming that the time delay of the target is acquired from the radar returns, two hyperbolic curves of the target can be obtained according to the layout position of the radar antennas. Figure 11 illustrates the ellipse cross positioning algorithm. Regardless of the effect of the wall, a Cartesian coordinate system is established with the center of the transmitting antenna. Supposing the target coordinates are O(x,y), the receiving antenna coordinates are (±d,0), and the time delays of the target to the two receiving antennas are $\tau_{1},\tau_{2}$. Then, according to the properties of the ellipse curves, the following equation holds:

(17) $$\begin{array}{l} OR_{1}+OT=c\times \tau_{1}=2a_{1}\\ OR_{2}+OT=c\times \tau_{2}=2a_{2}\\ b_{1}^{2}=a_{1}^{2}-{\left(\frac{d}{2}\right)}^{2}\\ b_{2}^{2}=a_{2}^{2}-{\left(\frac{d}{2}\right)}^{2} \end{array}$$

where c is the velocity of the electromagnetic waves; $2a_{1},2a_{2}$ are the major axis parameters; and $2b_{1},2b_{2}$ are the minor axis parameters of these ellipses’ curves. Based on these properties, the ellipses equations can be constructed:

(18) $$\left\{ \begin{array}{l} \frac{{\left(x-\frac{d}{2}\right)}^{2}}{{\left(\frac{c\tau_{1}}{2}\right)}^{2}}+\frac{y^{2}}{{\left(\frac{c\tau_{1}-d}{2}\right)}^{2}}=1\\ \frac{{\left(x+\frac{d}{2}\right)}^{2}}{{\left(\frac{c\tau_{2}}{2}\right)}^{2}}+\frac{y^{2}}{{\left(\frac{c\tau_{2}-d}{2}\right)}^{2}}=1 \end{array}\right.$$

**Figure 12:**
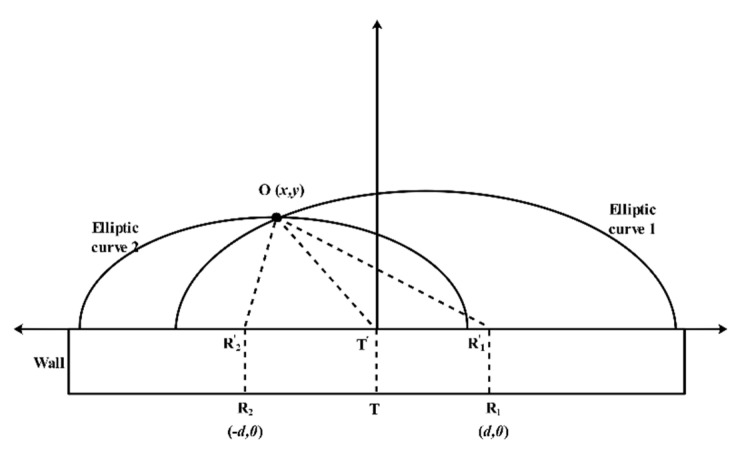
The illustration of the ellipse cross positioning algorithm (with obstacles between the radar and target).

The solution of (18) is the position of the target. When the radar and the target are obstructed by the wall, it is necessary to consider the wall’s physical parameters. If the thickness of the wall is not greater than 0.4 m, and the relative dielectric constant is small (not greater than 12), only the propagation delay of the electromagnetic wave in the wall should be considered, as shown in Figure 12. Therefore, the expression of (18) needs to be rewritten as follows:

(19) $$\begin{array}{c} OR_{1}+OT=c\times \left(\tau_{1}-2\times \frac{d_{\mathrm{wall}}}{\frac{c}{\sqrt{\varepsilon_{r}}}}\right)=2a_{1}\\ OR_{2}+OT=c\times \left(\tau_{2}-2\times \frac{d_{\mathrm{wall}}}{\frac{c}{\sqrt{\varepsilon_{r}}}}\right)=2a_{2}\\ b_{1}^{2}=a_{1}^{2}-{\left(\frac{d}{2}\right)}^{2}\\ b_{2}^{2}=a_{2}^{2}-{\left(\frac{d}{2}\right)}^{2} \end{array}$$

where $d_{wall}$ denotes the wall thickness, and $\varepsilon_{r}$ represents the permittivity of the wall.

**Figure 13:**
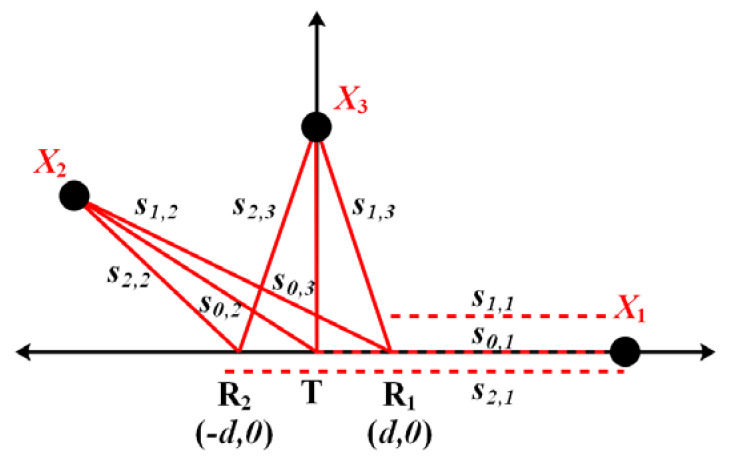
The scheme of the layout of the radar antennas and the targets in the radar power area.

The description of the localization principle shows that the time delay for all received channels needs to reach the match condition. The match condition means that the all-time delay is generated by the same target. The work in [55] discusses in detail the time delay match conditions of the target within the radar power range. Assuming that the target’s potential coordinates are $X_{1}\left(x_{1},0\right),X_{2}\left(x_{2},y_{2}\right),X_{3}\left(0,y_{3}\right)$, and that the distances between target and receiver are denoted by $s_{i,j},i=0,1,2;\;j=1,2,3$, where i represents the code of the receiver, j denotes the target number. Figure 13 shows the scheme of the layout of the radar antennas and the targets in the radar power area. Using the all-feasible cases of the target position $\left\{X_{1},X_{2},X_{3}\right\}$, the match condition can be determined.

Case 1, $X_{1}(x_{1},0)$. Due to the antenna’s hardware limitations, the target located in $X_{1}$ cannot be detected. For the sake of generality, we still take this target into consideration. Based on Figure 13, the time delay of $X_{1}$ can be expressed as follows:

(20) $$\begin{array}{c} \tau_{1,1}=\frac{s_{0,1}+s_{1,1}}{c}=\frac{2s_{1,1}+d}{c}\\ \tau_{1,2}=\frac{s_{0,1}+s_{2,1}}{c}=\frac{2s_{1,1}+3d}{c} \end{array}$$

Therefore, the time delay relations hold:

(21) $$|\tau_{1,2}-\tau_{1,1}|=\frac{2d}{c}$$

Case 2, $X_{2}(x_{2},y_{2})$. The time delays for the two receiving channels are as follows:

(22) $$\begin{array}{c} \tau_{2,1}=\frac{s_{0,2}+s_{1,2}}{c}\\ \tau_{2,2}=\frac{s_{0,2}+s_{2,1}}{c} \end{array}.$$

In this case, the distance of the transmitter-target-receiver satisfies the triangle theorem, so the two-channel time delay relationships are

(23) $$|\tau_{2,2}-\tau_{2,1}|=|\frac{\left|s_{2,2}-s_{1,2}\right|}{c}|\leq \frac{2d}{c}.$$

Case 3, $X_{3}(0,y_{3})$. The time delays for the two receiving channels are as follows:

(24) $$\begin{array}{c} \tau_{3,1}=\frac{s_{0,3}+s_{1,3}}{c}\\ \tau_{3,2}=\frac{s_{0,3}+s_{2,3}}{c} \end{array}.$$

Therefore, the time delay relations hold:

(25) $$|\tau_{3,2}-\tau_{3,1}|=0.$$

In summary, in the elliptical cross positioning algorithm, the upper limit of the delay difference of the same target in the radar receiving channel is related to the distance of the radar transmitting and receiving antenna and must be less than or equal to 2d/c.

### 4.2. Moving Human Detection

After the pre-processing and localization methods are introduced, the pre-processing method can improve the time delay (or termed time of arrival (TOA)) to obtain greater accuracy, and the correct TOA will ensure the accuracy of the positioning. Generally, the acquisition of TOA requires target detection operations. The detection method determines whether a target is absent or present in the examined radar signals based on statistical decision theory.

In this section, three categories of target detection methods used for UWB through-wall radar are reviewed. The first detection method is based on constant false rate (CFAR) detection, which can provide the maximum probability of detection under a given false alarm rate. The most common CFAR detectors [56] include cell averaging CFAR (CA-CFAR), cell averaging with greatest CFAR (CAGO-CFAR), and ordered statistics (OS-CFAR). The second detection method mainly uses the statistical characteristics of the received signal like skewness [57], kurtosis [58], standard deviation [59,60], variance [61], entropy [62], energy [63,64,65], etc. The third detection method is based on the multi-path model of the radar return signal. The CLEAN algorithm and its modified version are often used to detect the target and record the corresponding TOA.

**Figure 14:**
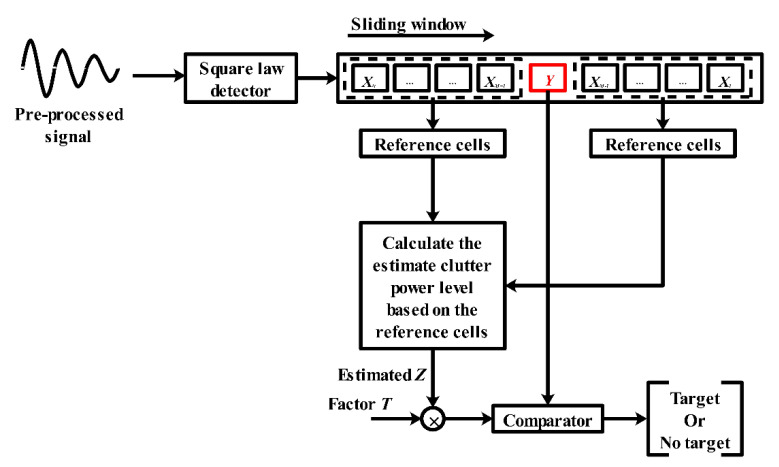
The scheme of the CFAR detector.

The CFAR is a sub-optimum detector widely used in through-wall radar. Figure 14 describes the general scheme of the CFAR detector. For a signal output by the square law detector, a sliding window is used to compare the signal power levels. The sliding window is composed of reference cells and test cells. The data span in the reference cells is employed to estimate the clutter power level. In Rohling [56], the random variables of the clutter contained in reference $X\in X_{1},X_{2},\ldots ,X_{N}$ were assumed to follow the exponential distribution. The probability density function could then be expressed as follows:

(26) $$p(x)=\{\begin{array}{l} \left(\frac{1}{\alpha^{2}}\right)e^{\left(-\frac{x}{\alpha^{2}}\right)},\;x\geq 0\\ 0,\;\mathrm{otherwise} \end{array}$$

where $\alpha^{2}$ denotes the clutter power level. Then, the probability of the false alarm $P_{fa}$ can be obtained as

(27) $$P_{fa}=\int_{S}^{\infty }p(x)dx$$

where S represents the threshold, which can be denoted by T×Z, where $Z=\alpha^{2}$. Under different false alarm probabilities, the T can be calculated by the following expression:

(28) $$T=\frac{1}{ln(P_{fa})}$$

Due to the different calculation methods used for Z, the traditional CFAR is derived from a variety of algorithms. In the case of CA-CFAR, the clutter power level is estimated as follows:

(29) $$Z=\frac{1}{N}\sum_{i=1}^{N}X_{i}$$

For CAGO-CFAR, the expression of Z is as follows:

(30) $$Z=\max\left(\frac{2}{N}\left[\sum_{i=1}^{N/2}X_{i}\right];\frac{2}{N}\left[\sum_{i=(N/2)+1}^{N}X_{i}\right]\right).$$

For OS-CFAR, unlike CA-CFAR, the calculation method of Z does not depend on the average of the clutter power level within the reference cells. Instead, the OS-CFAR sorts the clutter within the reference range by its amplitude and forms a new sequence:

(31) $$X_{\left(1\right)}\leq X_{\left(2\right)}\leq \cdots \leq X_{\left(N\right)}.$$

Therefore, the clutter power level is estimated by the ordered statistic $X_{\left(k\right)}$ instead of the arithmetic mean. The corresponding $P_{fa}$ for exponentially distributed clutter is obtained by the following iteration:

(32) $$P_{fa}=k\left(\begin{array}{l} N\\ k \end{array}\right)\frac{(k-1)!(T+N-k)!}{(T+N)!}.$$

Generally, k=N/2 is considered to easily calculate the threshold. The following equation expresses the relation between T and a given $P_{fa}$ under N reference cells:

(33) $$\prod_{i=N}^{(N/2)+1}\left(T+i\right)-\frac{N!}{P_{fa}\left(\frac{N}{2}\right)!}=0$$

**Figure 15:**
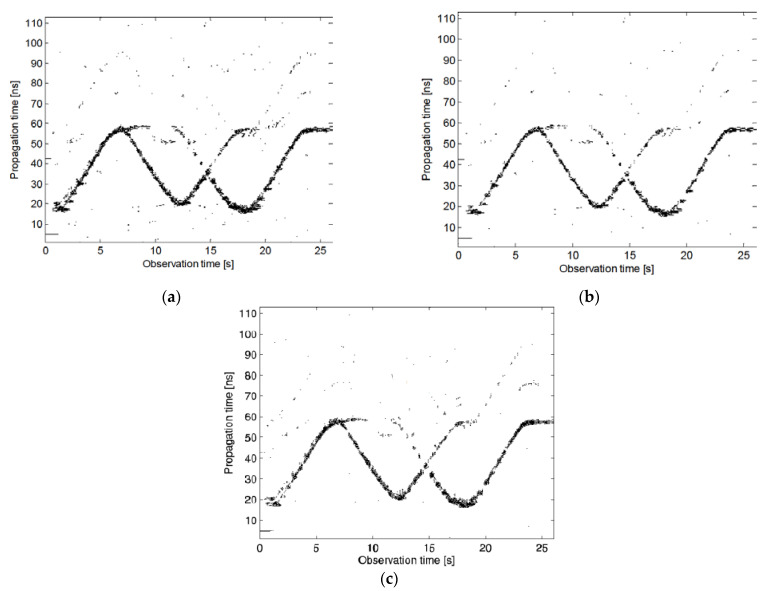
The detector result of CA-CFAR, CAGP-CFAR, and OS-CFAR. (**a**) CA-CFAR; (**b**) CAGO-CFAR; (**c**) OS-CFAR [66].

In Urdzik et al. [66], the performance of a variety of CFAR detectors is analyzed based on actual data measured by the UWB through-wall radar. In the experimental scene, two humans walk back and forth along the radar’s line of sight in an indoor gymnasium, and there is a wooden wall with a thickness of 24 cm between the radar and these human targets. Figure 15 shows the detection results of CA-CFAR, CAGO-CFAR, and OS-CFAR. In a detection scene with multiple human targets, the CA-CFAR and CAGO-CFAR detectors miss the detection of distant targets because the distant targets are covered by nearby targets, and the OS-CFAR has greater advantages in multi-target scenarios. Compared with Figure 15a–c is more adequate for the target trajectory because in the estimation of the clutter power level, the ordered statistics are selected as the noise estimation value, which can suppress the performance degradation caused by the shadowing effect to a certain extent [67].

The second method mainly focuses on the features of the signal along the fast-time dimension. Considering that the physical size of the human target has a certain thickness, the scattered signal of a human is always distributed in several neighboring bins. Therefore, based on the fast-time dimension of the echo, some potential features have different measurements between the target area and the non-target area. Using this parameter, the human target can be detected by setting the reference threshold.

**Figure 16:**
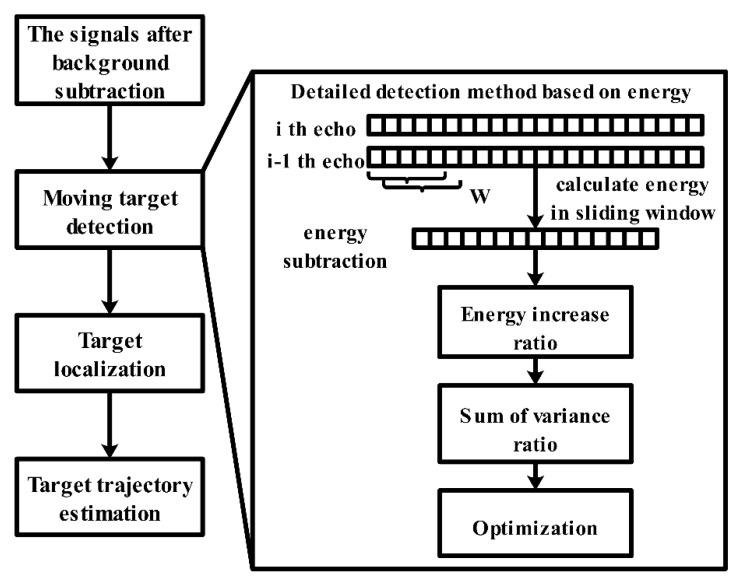
A scheme of the signal processing steps and detailed detection flow.

In Wu et al. [63], an efficient method for moving target detection and TOA estimation using UWB through-wall radar was proposed. The essence of this method is to change the signal energy in different regions. Figure 16 presents an overview of the signal processing steps and the detailed detection method based on energy. As the figure shows, the energy of the received signal is calculated in the sliding window section first. Then, the energy increase ratio is obtained and summed; next, we must find the index of the maximum sum sequence, where the index refers to the target’s time delay point. Finally, an optimization operation is used to check the time delay sequence that can reject the false estimated position and localize the target more correctly. This method requires a signal with a good signal–noise ratio (SNR). However, not all signals can guarantee a high SNR. This method provides the correct mechanism to handle this problem based on the previous estimation. Thus, the first estimated position is fundamentally important. The experimental results illustrate that the proposed method can effectively detect a moving target behind a wall and extract the trajectory.

**Figure 17:**
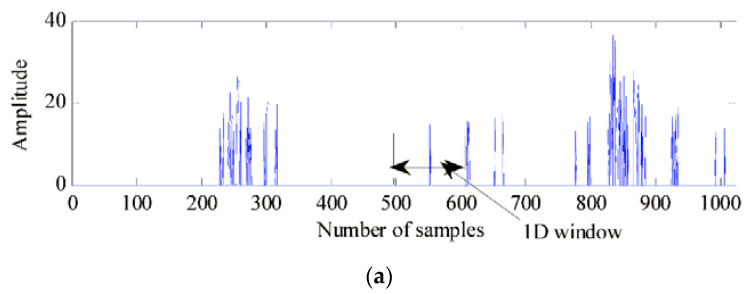

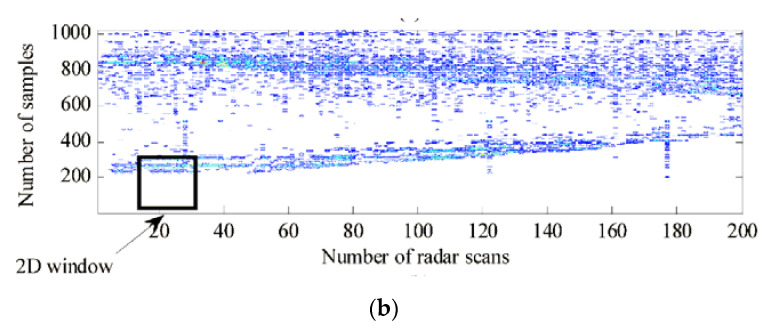
Jumping window method for eliminating false alarms: (**a**) 1D window and (**b**) 2D window [68].

**Figure 18:**
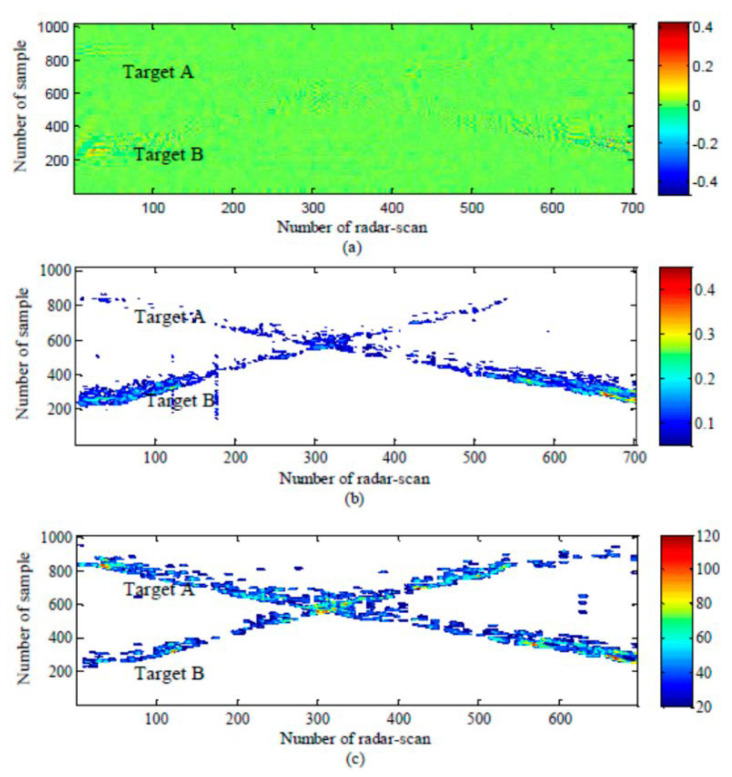
Radargrams: (**a**) before detection, (**b**) after detection with the conventional CLEAN algorithm, and (**c**) after detection with the modified CLEAN algorithm [68].

Since the UWB radar received signal can be modeled as a specular multipath model (see the Equation (2)), the human target echo is considered to be composed of signal components of several scattering paths that have range scalability. Therefore, in [21,22], the CLEAN algorithm was used for target detection to extract the time delay clutter generated by the target; the algorithm’s steps are introduced in Algorithm 3. The principle of CLEAN is to search for all pulses through the cross-correlation of the echo signal and the template signal. The final time delay is filtered by a pre-settled threshold. However, this algorithm is not suitable for long-distance target detection because the fixed threshold causes weak and distant targets to be ignored. This method is thus inappropriate to detect the target echo intensity under a fixed threshold. To address this problem, a modified CLEAN algorithm was proposed in [68]. Based on the conventional CLEAN algorithm, the weak signal compensating method and the multi-dimension jumping window were used. To reduce false alarms caused by signal compensation, 1D and 2D jumping windows were designed, as shown in Figure 17, Figure 18 provides a detection performance comparison between the conventional CLEAN algorithm and the modified CLEAN algorithm. In Liu [67], the detection performance of the CA-CFAR, OS-CFAR, and CLEAN algorithms was compared based on actual radar data, and the applicable environments and limitations were also discussed. Although OS-CFAR offers better performance in strong clutter and multi-path environments than CA-CFAR, both of them cannot detect a range-extended target in an ideal state. The CLEAN algorithm offers better performance for targets in different motion states and can effectively suppress clutter, multipath, and target occlusion, even when the targets are very close or overlapping.
**Algorithm 3** The steps of the CLEAN algorithm**Input:**The waveform shape s(t)The detection threshold $T_{clean}$**Output:**The estimated amplitude ${\hat{a}}_{i}(t)$The estimated time delay ${\hat{n}}_{i}(t)$1. Set the initial residual waveform $d_{0}(t)=w(t)$ and the initial counter i=0;2. Calculate the cross-correlation $r_{sd}(\tau )$ between s(t) and $d_{i}(t)$; the time-index associated with the maximum amplitude of $r_{sd}(\tau )$ is the $i_{th}$ estimated TOA:${\hat{n}}_{i}(t)=argmax_{\tau }|r_{sd}(\tau )|$.The cross-correlation at ${\hat{n}}_{i}(t)$ is the $i_{th}$ estimated amplitude:${\hat{a}}_{i}(t)=r_{sd}({\hat{n}}_{i}(t))$.If the path magnitude ${\hat{a}}_{i}(t)$ below the detection threshold is $T_{clean}$, stop and record the TOA and amplitude.3. Increase the iteration counter: i←i+1.4. Update the residual waveform:$d_{i}(t)=d_{i-1}(t)-{\hat{a}}_{i}(t)s(t-{\hat{n}}_{i}(t))$.5. Iterate: Go to step.2.* The waveform shape $s(t)$ can be approximated by the radar transmitted signal in free space or an anechoic chamber.**End**


### 4.3. Vital Sign Signal Measurement

Vital sign signal measurement based on UWB through-wall radar has great application potential because it does not require contact with the human target. For example, this technology can be used for human perception, positioning, and rescue under collapsed obstacles caused by earthquakes, mine disasters, or other disasters. Meanwhile, it can also be used for medical non-contact human parameter acquisition and monitoring. In cases like the outbreak of COVID-19, a non-contact human-parameter-obtaining method will help reduce the risk of infection among medical staff. As mentioned above, the IR-UWB through-wall radar has a high range resolution, allowing it to estimate the respiratory rate (RR) and heartbeat rate (HR) of a human target by detecting the chest contraction displacements caused by breathing and the heart position changes caused by heartbeats. Generally speaking [69], for normal adults, the RR and HR spectrum ranges in the frequency domain by about 0.2–0.3 Hz (12–20 beats/min) and 1–1.6 Hz (60–100 beats/min), respectively. For young children, the range of RR and HR is about 0.2–0.6 Hz (17–40 beats/min) and 1.2–3.2 Hz (70–190 beats/min), respectively. In particular, the RR and HR of a human buried by a disaster will fluctuate greatly. In Liu et al. [70], the spectrum distribution range of a respiratory signal (0.2–0.8 Hz) and heartbeat signal (1–2.5 Hz) of a buried human was considered.

**Figure 19:**
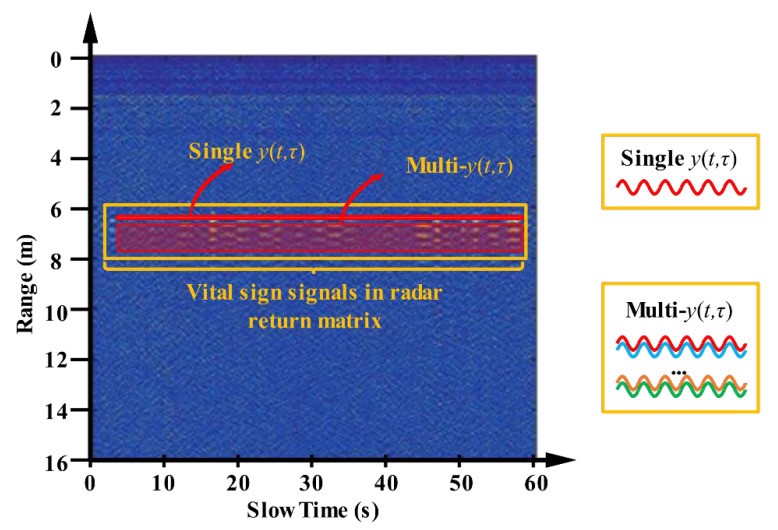
Vital sign signals in radar return matrix [25].

As mentioned before, in a vital signal measurement scene, the UWB radar return signal model using Equation (2) should be analyzed in the slow time dimension. By denoting as the radar return with respiratory rate and heartbeat information, the y(t,τ) belonging to the radar return matrix (see Figure 19, the single y(t,τ) means the single frame signal, and the multi-y(t,τ) means multiply frame signals) can be expressed as follows [71]:

(34) $$\begin{array}{ll} y(t,\tau ) & =Ap(\tau -\tau_{v}(t))\\ & =Ap(\tau -\tau_{0}-\tau_{r}sin(2\pi f_{r}t)\\ & -\tau_{h}sin(2\pi f_{h}t+\varphi_{h})) \end{array}$$

where $f_{r}$, $f_{h}$ represents the respiratory frequency and heartbeat frequency, respectively. Obviously, the spectrum analysis for y(t,τ) can obtain $f_{r}$, $f_{h}$. The Fourier transform (FT) is operated in slow time y(t,τ) as

(35) $$Y(f,\tau )=\int_{-\infty }^{+\infty }y(t,\tau )e^{-j2\pi ft}dt$$

Then, Y(f,τ) can be obtained from the 2D Fourier transform of y(t,τ) as y(f,ν):

(36) $$Y(f,\nu )=\int_{-\infty }^{+\infty }Y(f,\nu )e^{j2\pi \nu \tau }d\nu$$

After synthesizing (34) and (35), y(f,ν) can be represented as follows:

(37) $$\begin{array}{ll} y(f,\nu ) & =\int_{-\infty }^{+\infty }\int_{-\infty }^{+\infty }y(t,\tau )e^{-j2\pi ft}e^{-j2\pi v\tau }dtd\tau \\ & =\int_{-\infty }^{+\infty }\int_{-\infty }^{+\infty }AP(\nu )e^{-j2\pi ft}e^{-j2\pi v\tau_{d}(t)}dt\\ & =AP(\nu )e^{-j2\pi \nu \tau_{0}}\cdot \int_{-\infty }^{+\infty }\int_{-\infty }^{+\infty }e^{-j2\pi \nu m_{f}sin(2\pi f_{r}t)}\\ & \cdot e^{-j2\pi \nu m_{h}sin(2\pi f_{h}t+\varphi_{h})}e^{-j2\pi ft}dt \end{array}$$

where P(ν) is the FT of the received signal in fast time P(τ) based on the Bessel series [72]:

(38) $$e^{-jzsin(2\pi f_{0}t)}=\sum_{k=-\infty }^{+\infty }J_{k}(z)e^{-j2\pi kf_{0}t}$$

The Equation (37) can then be updated as

(39) $$\begin{array}{ll} Y(f,\nu ) & =AP(\nu )e^{-j2\pi v\tau_{0}}\\ & \cdot \int_{-\infty }^{+\infty }\left(\sum_{k=-\infty }^{+\infty }J_{k}(\beta_{r}\nu )e^{-j2\pi kf_{r}t}\right)\\ & \cdot (\sum_{k=-\infty }^{+\infty }J_{l}(\beta_{h}\nu )e^{-j2\pi lf_{h}t}e^{-j2\pi l\varphi_{h}t}e^{-j2\pi ft})dt \end{array}$$

where $\beta_{r}=2\pi m_{r}$ and $\beta_{h}=2\pi m_{h}$. Using (39) in (35), the frequency spectrum in slow time can be expressed as

(40) $$Y(f,\tau )=A\sum_{k=-\infty }^{+\infty }\sum_{l=-\infty }^{+\infty }e^{-jl\varphi_{h}}G_{kl}(\tau )\delta (f-kf_{r}-lf_{h})$$

where $G_{kl}$ is given by

(41) $$G_{kl}(\tau )=\int_{-\infty }^{+\infty }P(\nu )J_{k}(\beta_{r}\nu )J_{l}(\beta_{h}\nu )e^{j2\pi \nu (\tau -\tau_{0})}dv$$

Considering $\tau =\tau_{0}$, Equation (41) can then be maximized as

(42) $$G_{kl}(\tau_{0})=\int_{-\infty }^{+\infty }P(\nu )J_{k}(\beta_{r}\nu )J_{l}(\beta_{h}\nu )dv$$

Therefore, the final frequency spectrum expression in slow time can be expressed as follows:

(43) $$Y(f,\tau )=A\sum_{k=-\infty }^{+\infty }\sum_{l=-\infty }^{+\infty }e^{-jl\varphi_{h}}G_{kl}\delta (f-kf_{r}-lf_{h})$$

Equation (43) contains the respiratory frequency, the heartbeat frequency, and the corresponding harmonic frequency. It is clear that the spectrum here is a discrete function and that its amplitude depends on $G_{kl}$.

**Figure 20:**
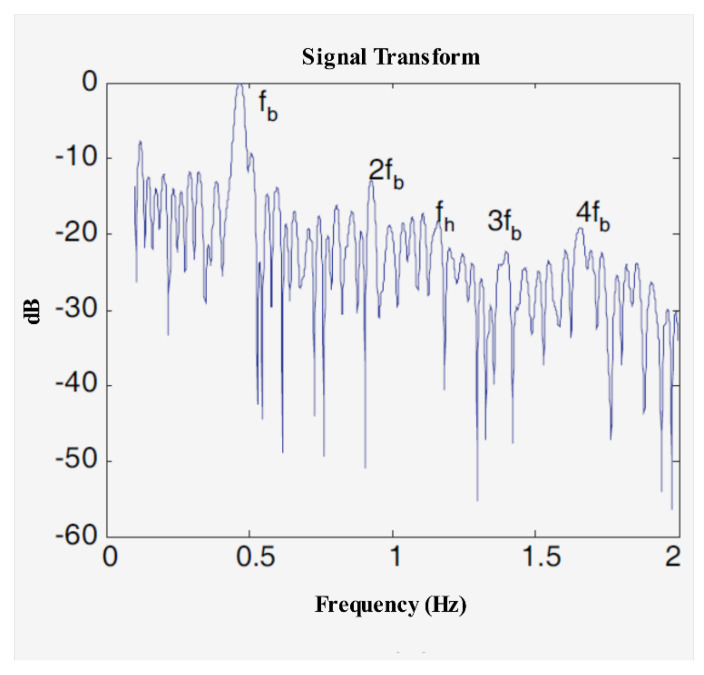
The breathing signal and heart signal normalized spectrum in the frequency domain [71].

HR and RR have different and non-overlapping frequency bands, so it is feasible to find the RR from the spectrum. However, the multiple order harmonics of respiratory signals may appear in the frequency of HR, and their amplitudes may be even larger than that of the HR. The research in [71] showed that the fourth harmonic and the third-order intermodulation waveform of a respiratory signal is equivalent to the amplitude of a heartbeat signal under a through-wall condition (see Figure 20). Therefore, to obtain HR and RR more accurately, it is necessary not only to improve the frequency resolution, but also to suppress the multiple harmonics of the respiratory signal.

**Figure 21:**
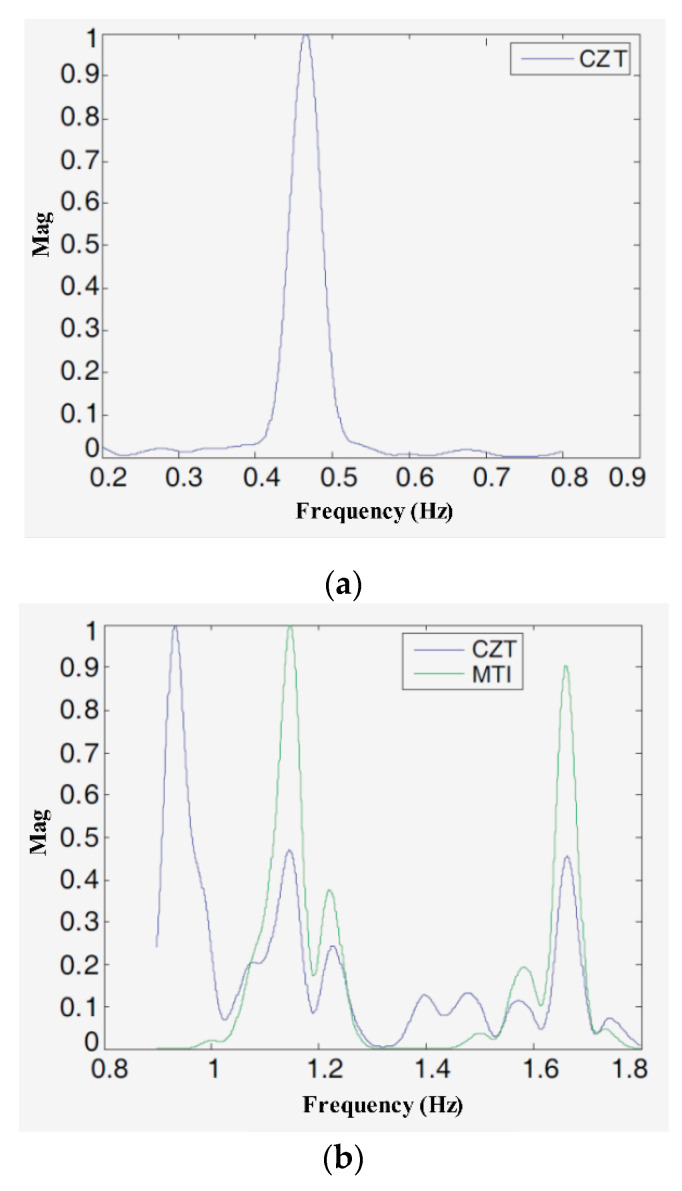
The real-world results [71]. (**a**) The breathing signal frequency spectrum based on CZT; (**b**) The heartbeat signal frequency spectrum based on CZT and MTI.

In Lazaro et al. [71], the harmonics of a respiratory signal and the intermodulation effects between the respiratory signal and the heartbeat signal were analyzed in detail. This study also showed that when the respiratory displacement is greater than 2.5 mm, the magnitude of these respiratory harmonics is the same as that of the heartbeat signal. Meanwhile, the paper concluded that the transmitted pulse shape has an important influence on the harmonic’s components and that, under a given central frequency and bandwidth, the ratio between the fundamental frequency components of the respiratory rate and heartbeat is independent of the transmitted pulse shape. To improve the spectrum resolution, the author used Chirp Z-transform (CZT) instead of fast Fourier transform (FFT) and designed a harmonic canceller based on a moving target indicator (MTI) to suppress the respiratory harmonics. The real-world experience results showed that the proposed method can effectively remove the effects of harmonics. As Figure 21 shows, with the MTI harmonic canceller and CZT, the heartbeat frequency can be determined easily.

**Figure 22:**
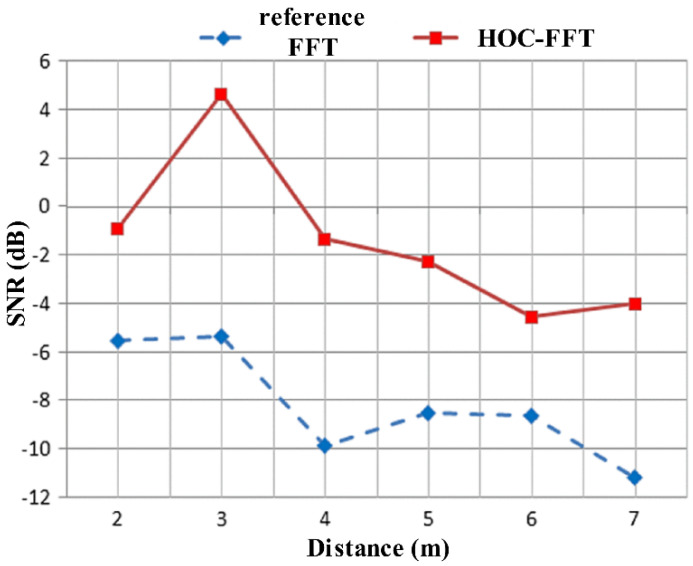
The SNR changes in different distances between the reference FFT and HOC-FFT.

**Figure 23:**
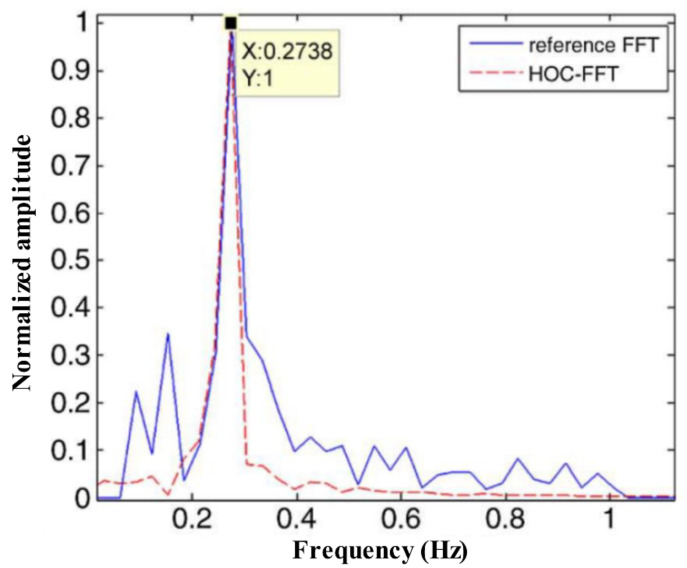
The experiment respiration frequency result between reference FFT and HOC-FFT.

Because the echo carrying human life information has quasi periodicity in slow time, a novel detection method for radar vital sign signals based on a high-order cumulant (HOC) was proposed in [73]. Since the theoretical value of the high-order statistics of Gaussian noise is zero, this method is insensitive to Gaussian noise. The SNR of the signal also effectively improved via the HOC, as shown in Figure 22. The simulation and experimental results show that compared to traditional FFT, the proposed method has the advantages of simplicity, high output SNR, and high-order harmonic suppression. The experimental results are shown in Figure 23.

The FFT method assumes a stationary signal, so it is not suitable for life signal processing. Some scholars use time-frequency analysis methods to process life signals, including short-time Fourier transform (STFT) [74], two-dimension Fourier transform (2D-FFT) [74], wavelet transform (WT) [75,76], Hilbert–Huang transform (HHT) [70,77,78], etc. In Li et al. [74], 2D-FFT, S transform (ST) was used to analyze the respiratory frequency characteristics of a human behind a wall in three different cases (i.e., bradypnea, eupnea, and tachypnea; the corresponding frequencies were about 0.25, 0.35, and 0.7 Hz, respectively).

In Liu et al. [70], the characteristics of thoracic movement caused by human respiration were analyzed using numerical simulations and measured data. HHT was used to successfully identify and distinguish the respiratory characteristics of different testers in various breathing states (i.e., normal breath, holding breath, and repeating ‘123’). Due to the limitations of the hardware resolution of through-wall radar, a high center frequency is required for reliable respiration and heart rate extraction. Based on the finite difference time domain (FDTD) numerical analysis method, a human body simulation model of UWB radar under the conditions of collapsed buildings after an earthquake was established in [77]. In this model, humans are buried in ruins in different positions and have different vital signs. The experimental and simulation results show that a time–frequency transform based on HHT can effectively obtain the respiration rate of a human body. However, weak heartbeat information cannot be effectively extracted. Based on the empirical mode decomposition (EMD) algorithm, the radar echo signal of the target was adaptively decomposed into multiple intrinsic mode functions (IMFs) in [79]. By analyzing and calculating the energy spectrum characteristics of each IMF, the respiratory and heartbeat signals were reconstructed in the time domain. In Zhang et al. [80], on the basis of [79], the ensemble empirical mode decomposition (EEMD) algorithm was used to suppress the mode aliasing in EMD and improve the measurement accuracy. In Yan et al. [58], the variable mode decomposition (VMD) algorithm was used to suppress the modal aliasing problem. VMD can accurately estimate the heart rate information of multiple targets behind the wall. However, the key parameters of the VMD decomposition level should be determined according to the number of targets in the detection scene, which limits this method’s application. The above studies did not consider the impact of random body movements (RBMs) [81] during the vital sign parameter measurement process. In Lazaro et al. [82], respiratory movement and RBMs were divided into micro-motion and macro motion, respectively. A time delay threshold was defined to detect stationary and non-stationary humans. If the human standard deviations of time delay exceed a certain threshold, the RR and HR during the period will be neglected. However, this paper did not note that arm swings or speaking could also have an impact on the vital sign parameter measurement results. In Khan et al. [69], the auto-correlation method was proposed to detect and remove the RBM part to reduce the errors from the heartbeat rate and respiratory rate measurements. Meanwhile, the proposed method was also found to be useful for small RBMs like speaking, shaking one’s head slightly, or moving one’s body slightly.

**Figure 24:**
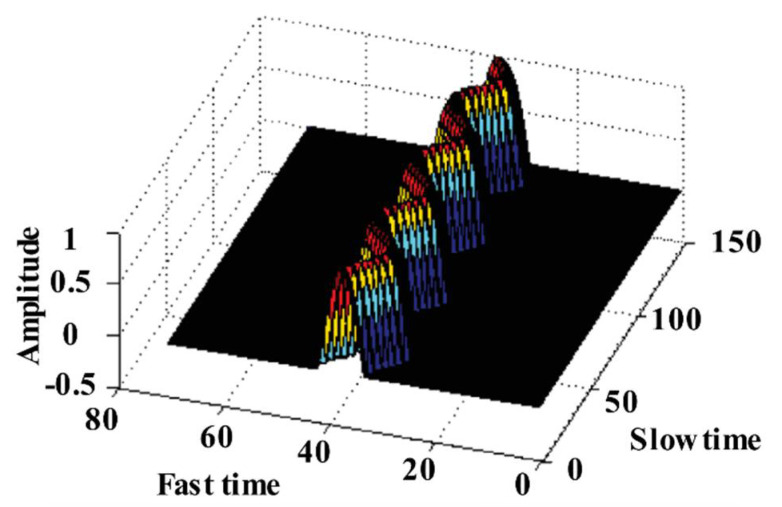
The banded signal of human breathing.

In previous related studies, human heart rates and respiratory rates were mainly analyzed using single frame UWB radar echoes to find the relevant data frame in the radar return matrix. Indeed, the human return waves present range expansion in UWB radar detection. Therefore, the echo signal carrying breathing and heartbeat information can be regarded as a banded signal in space, shown in Figure 19 and Figure 24. Based on this core idea, the sliding time domain window is proposed to accumulate multi-frame data to improve the signal SNR and shorten the observation times for humans [83]. In Yang et al. [84], a similar processing method based the multi-frame data is used to improve the signal SNR.

**Figure 25:**
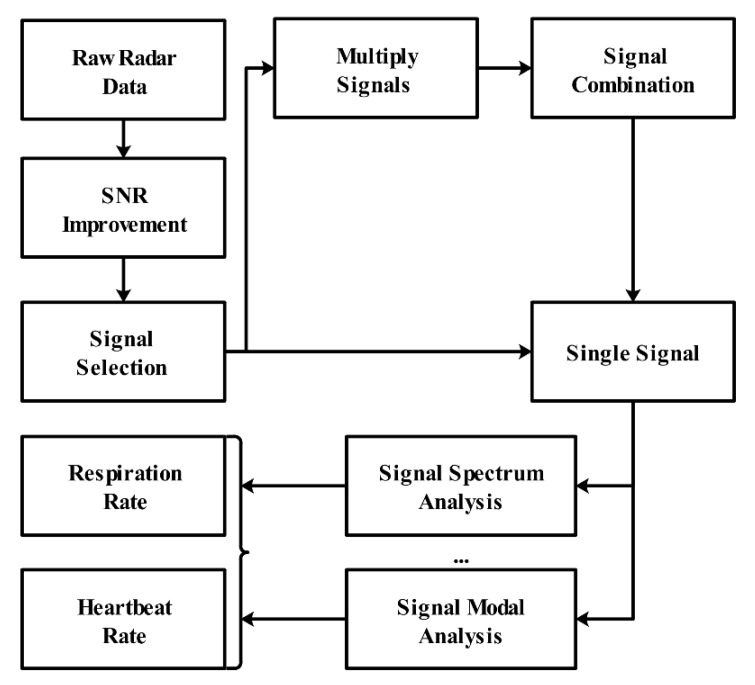
The vital sign signal processing framework using UWB radar.

Generally speaking, for the vital sign signal measurement using UWB through-wall radar, the processing framework can be summarized as the following Figure 25.

## 5. Conclusions and Future Prospects

Ultra-wideband technology has played a huge role in life rescue, anti-terrorism, and other fields. In this paper, we reviewed the common technological methods used in the UWB through-wall radar and introduced the generalized IR-UWB radar components and their development history. The general UWB radar return signal model was also introduced based on the UWB signal channel. We hope that this article will not only provide meaningful references for researchers in this field, but also provide some feasible ideas for the development of ultra-wideband radar products and technologies.

Although the use of the UWB through-wall radar has made great progress, we hope that the remaining problems can be solved in the future:

(1) Research on the cognitive detection of penetrating media. The most important task of through-wall radar is to detect a target behind non-metallic obstacles, as the physical characteristics of obstacles lead to target position distortion. At present, the parameter estimation method for through-wall radar is mainly based on SFCW UWB radar. For IR-UWB through-wall radar, determining how to recognize the parameters of obstacles in real-time is a problem worthy of further study.
(2) Research on the UWB radar general integrated system. According to the particularities of the scene used by UWB radar, in addition to through-wall radar, ground penetrating radar and life detector radar can also be used. In fact, the design architectures of these three radar types are largely the same, and their signal processing algorithms also tend to be the same. Therefore, it should be determined whether these three kinds of radar can be integrated in a general system. Based on cognitive radar theory and artificial intelligence, a general system can be enabled to complete adaptive optimal detection based on a complex real-world environment.
(3) Research on environment modeling. At present, many UWB through-wall radar systems have only been tested under ideal experimental conditions. However, if applied in real-world scenes, such radar solutions may face many unpredictable problems. Therefore, the gap between real-world and lab environments is worthy of attention.
(4) Research on UWB radar networking. The performance of a single radar is limited in complex environments. Multiplying through-wall radar could effectively enhance the ability of environmental perception and target information acquisition. Thus, the protocol of radar networking needs to be studied.

## Figures and Tables

**Table 1:** Error comparisons of the proposed method and the ESPRIT method.

	Thickness (cm)		Relative Permittivity		Conductivity (S/m)	
	Actual	Error	Actual	Error	Actual	Error
Proposed method	10	2.2%	6	3.8%	0.01	15.5%
ESPRIT method		19.5%		66.7%		40.7%
Proposed method	20	0.39%		0.5%		20.4%
ESPRIT method		3.15%		6.2%		24.1%
Proposed method	30	0.07%		0.33%		22.9%
ESPRIT method		0.67%		1%		21.8%

**Table 2:** Estimated wall parameters under various SNR levels.

SNE(dB)	Thickness (cm)		Relative Permittivity		Conductivity (S/m)	
	Estimated	Actual	Estimated	Actual	Estimated	Actual
0	20.73	20	5.60	6	0.0048	0.01
5	20.33		5.83		0.0068	
10	19.65		6.22		0.0085	
15	19.83		6.11		0.0112	
20	20.19		5.91		0.0108	
25	20.08		5.97		0.0110	
30	20.08		5.97		0.0108	

## Data Availability

The data presented in this study are available on request from the corresponding author. The data are not publicly available due to privacy.
